# Lipoprotein (a) interactions with cholesterol-containing lipids on angiographic coronary collateralization in type 2 diabetic patients with chronic total occlusion

**DOI:** 10.1186/s12933-019-0888-z

**Published:** 2019-06-24

**Authors:** Ying Shen, Shuai Chen, Yang Dai, Xiao Qun Wang, Rui Yan Zhang, Zhen Kun Yang, Jian Hu, Lin Lu, Feng Hua Ding, Wei Feng Shen

**Affiliations:** 1 Department of Cardiology, Rui Jin Hospital, Shanghai Jiao Tong University School of Medicine, Shanghai, 200025 People’s Republic of China; 2 Institute of Cardiovascular Diseases, Shanghai Jiao Tong University School of Medicine, 197 Rui Jin Road II, Shanghai, 200025 People’s Republic of China

**Keywords:** Lipoprotein (a), Low-density lipoprotein, Non-high-density lipoprotein cholesterol, Coronary collateral circulation, Stable coronary artery disease

## Abstract

**Background:**

We investigated whether or to what extent the interaction of lipoprotein (a) [Lp(a)] with cholesterol-containing lipids was associated with angiographic coronary collateralization in type 2 diabetic patients with chronic total occlusion.

**Methods:**

Serum levels of Lp(a), total cholesterol, low-density lipoprotein–cholesterol (LDL-C), high-density lipoprotein–cholesterol (HDL-C), and triglyceride were determined and non-HDL-C was calculated in 706 type 2 diabetic and 578 non-diabetic patients with stable coronary artery disease and angiographic total occlusion of at least one major coronary artery. The degree of collaterals supplying the distal aspect of a total occlusion from the contra-lateral vessel was graded as poor (Rentrop score of 0 or 1) or good coronary collateralization (Rentrop score of 2 or 3).

**Results:**

For diabetic and non-diabetic patients, Lp(a), total cholesterol, LDL-C, and non-HDL-C levels were higher in patients with poor coronary collateralization than in those with good collateralization, whereas HDL-C and triglyceride levels were similar. After adjustment for potential confounding factors, tertiles of Lp(a), total cholesterol, LDL-C and non-HDL-C remained independent determinants for poor collateralization. A significant interaction between Lp(a) and total cholesterol, LDL-C or non-HDL-C was observed in diabetic patients (all P interaction < 0.001) but not in non-diabetics. At high tertile of total cholesterol (≥ 5.35 mmol/L), LDL-C (≥ 3.36 mmol/L) and non-HDL-C (≥ 4.38 mmol/L), diabetic patients with high tertile of Lp(a) (≥ 30.23 mg/dL) had an increased risk of poor collateralization compared with those with low tertile of Lp(a) (< 12.66 mg/dL) (adjusted OR = 4.300, 3.970 and 4.386, respectively, all P < 0.001).

**Conclusions:**

Increased Lp(a) confers greater risk for poor coronary collateralization when total cholesterol, LDL-C or non-HDL-C are elevated especially for patients with type 2 diabetes.

**Electronic supplementary material:**

The online version of this article (10.1186/s12933-019-0888-z) contains supplementary material, which is available to authorized users.

## Background

Abundant evidence suggests that in the case of atherothrombotic coronary artery obstruction, coronary collateral formation compensates myocardial ischemia [1, 2] and improves patients’ clinical outcome and even survival [3, 4]. The mechanism of collateral vessel growth is complex involving arteriogenesis which pertains to the remodeling of preexisting arterial vessels through the anatomic increase in lumen area and wall thickness and angiogenesis defined as new capillaries that stem from the budding of preexisting capillary vessels. Arteriogenesis is likely caused by a combination of mechanical (shear stress) and chemical factors (related to ischemia and genes activated by ischemia), whereas angiogenesis is thought to be related to tissue hypoxia and the chemical factors produced under these conditions [2, 3]. These processes of growth and maturation of coronary collateral vessels are also influenced by multiple clinical and biochemical factors, inflammatory cytokines, and growth factors [5–9].

Lipoprotein (a) [Lp(a)] which is genetically determined contains principally a cholesterol rich low-density lipoprotein particle, one molecule of apolipoprotein (apo) B-100 and an apo (a) [10], and represents an atherothrombogenic lipoprotein [11]. Although the distribution of serum Lp(a) levels is very skewed, elevated circulating Lp(a) has emerged as an independent and causal cardiovascular risk factor and an important predictor of adverse outcomes for both general and higher risk populations [12], especially when low-density lipoprotein cholesterol (LDL-C) levels are elevated [13]. Previous studies with a small sample size have suggested an inverse relation between serum levels of Lp(a) and development of coronary collateral circulation [14, 15], with high levels of Lp(a) associated with reduced production and bioactivity of vascular endothelial growth factor [14, 16]. Hypercholesterolemia particularly with high levels of LDL-C and/or low levels of high-density lipoprotein cholesterol (HDL-C) is an established coronary risk factor that induces endothelial cell dysfunction and impairs collateral vessel growth [17]. Non-high-density lipoprotein cholesterol (non-HDL-C)—the sum of cholesterol in other lipoproteins except for high-density lipoprotein—is closely associated with coronary atheroma progression and cardiovascular outcome, and has been proposed to improve risk estimation beyond total cholesterol and/or LDL-C [18, 19], especially for individuals with LDL-C levels that are not high or have already reached the treatment goal when the triglyceride level is elevated [20].

Diabetes mellitus represents a powerful independent risk factor for increased cardiovascular mortality associated with coronary artery disease, partly because of an impaired physiological adaptive response of coronary collateral circulation [5, 7]. Hyperglycemia induces microvascular rarefaction in the myocardium even without ischemia, and capillary density further decreased in chronic ischemia hearts [21]. Patients with type 2 diabetes often have substantially adverse functional and structural remodeling of the coronary arterioles and even amongst those without known coronary artery disease, suggesting that diabetes may destabilize microvascular vessels of the heart and impair the responsiveness of ischemic myocardium to pro-angiogenic factors [22, 23]. However, the impact of plasma lipid levels on coronary collateralization in diabetes remains unclear. In this study, we hypothesized that the interaction of genetic lipoprotein (a) [Lp(a)] with environmental cholesterol-containing lipids (i.e., total cholesterol, LDL-C, and non-HDL-C) is associated with angiographic coronary collateralization in type 2 diabetic patients with stable coronary artery disease and chronic total occlusion.

## Methods

### Study population

**Fig. 1:**
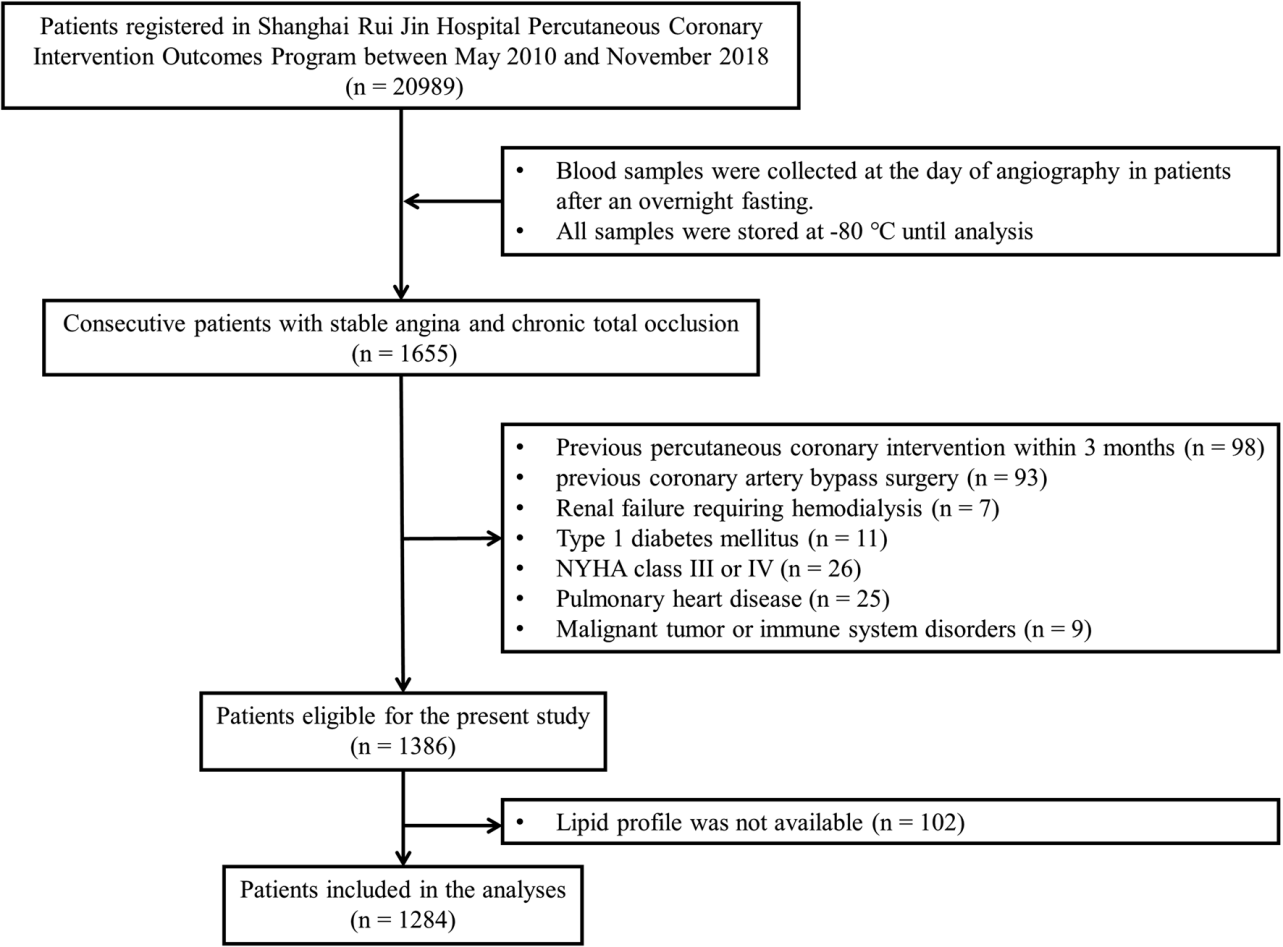
Flowchart of patient enrollment

A total of 1655 consecutive patients with stable coronary artery disease and chronic total occlusion (> 3 months) of at least one major epicardial coronary artery between May 2010 and November 2018 were screened from the database of Shanghai Rui Jin Hospital PCI Outcomes Program. This angiographic inclusion criterion of study patients was used because a severe coronary artery obstruction was a prerequisite for spontaneous collateral recruitment [4]. The duration of coronary artery occlusion was estimated from the date of occurrence of myocardial infarction in the area of myocardium supplied by the occluded vessel, from an abrupt worsening of existing angina pectoris, or from information obtained from a previous angiogram. For the purpose of this study, 258 patients were excluded because of PCI within the last 3 months (n = 98), a history of coronary artery bypass grafting (CABG) (n = 93), renal failure requiring hemodialysis (n = 7), chronic heart failure with NYHA class III or IV (n = 26), pulmonary heart disease (n = 25) and malignant tumor or immune system disorders (n = 9), as these conditions could influence collateral formation. Patients with type 1 diabetes (n = 11) were excluded by measurement of C-peptide level [5]. One hundred and two patients were further excluded due to unavailability of lipid profile. Thus, the remaining 1284 patients were enrolled in the final analyses. Among them, 706 patients had type 2 diabetes and 578 were non-diabetics (Fig. 1).

The diagnosis of type 2 diabetes was made according to the criteria of the American Diabetes Association, including glycosylated hemoglobin (HbA1c) ≥ 6.5%, fasting plasma glucose concentration ≥ 7.0 mmol/L, 2-h postprandial glucose concentration ≥ 11.1 mmol/L, or a random plasma glucose ≥ 11.1 mmol/L in a patient with classic symptoms of hyperglycemia or hyperglycemic crisis [24]. Hypertension was defined as systolic blood pressure ≥ 140 mmHg and/or diastolic blood pressure ≥ 90 mmHg, or use of anti-hypertensive agents for controlling blood pressure [25]. Dyslipidemia was defined according to the Third Report of The National Cholesterol Education Program (NCEP) [26]. Stable angina was diagnosed according to the criteria recommended by the American College of Cardiology/American Heart Association [27].

The study protocol was approved by the Institutional Review Board of Rui Jin Hospital, Shanghai Jiaotong University School of Medicine and clinical investigation was conducted according to the principle of the Declaration of Helsinki. Written informed consent was obtained from all patients.

### Biochemical investigation

Blood samples were obtained at the day of angiography in all patients after an overnight fasting. Serum levels of creatinine, lipid profiles (including triglyceride, total cholesterol, LDL-C, HDL-C, and non-HDL-C), glucose, and glycosylated hemoglobin (HbA1c) were determined with standard laboratory techniques [7–9]. Glomerular filtration rate (GFR) was estimated using the Chronic Kidney Disease Epidemiology Collaboration (CKD-EPI) equation: GFR_EPI_ (mL/min/1.73 m^2^) = 141 × min (creatinine/k, 1)^α^ × max (creatinine/k, 1)^−1.209^ × 0.993^age^ × 1.018 [if female], where k is 0.7 for females and 0.9 for males, α is − 0.329 for females and − 0.411 for males, min indicates the minimum of creatinine/k or 1, and max indicates the maximum of creatinine/k or 1 [28]. Serum Lp(a) and high-sensitivity C-reactive protein (hsCRP) levels were assayed by ELISA (Biocheck Laboratories, Toledo, OH, USA).

### Angiography and collateral grading

Coronary angiography was performed through the femoral or radial access with 6 Fr diagnostic catheters. All angiograms were analyzed independently by two blinded interventional cardiologists. The degree of coronary artery disease was assessed according to lesion classification scheme of the American College of Cardiology/American Heart Association [29]. The presence and degree of coronary collateralization from the contra-lateral vessel (often via connections of the epicardial surface or intraventricular septum) was visually estimated using the Rentrop scoring system,as this angiographic assessment of coronary collaterals is routinely applied in clinical practice [7–9, 30]. In patients with more than one chronic total occlusion, the vessel with the highest collateral grade was chosen for analysis. In case of disagreement, the difference in interpretation was resolved by a third reviewer.

### Statistical analysis

Data are presented as mean ± standard deviation (SD) and number (percentages). For continuous variables, differences between groups were evaluated by t test for normally distributed values; otherwise, the Mann–Whitney U test was applied. For categorical variables, differences between groups were evaluated with the Chi-square test. To examine the relationships between Lp(a) and other cholesterol-containing lipid profiles, we employed Pearson’s correlation. The serum levels of Lp(a) and lipid profile (total cholesterol, LDL-C, non-HDL-C, HDL-C, triglyceride) were divided into 3 groups according to tertile distribution, respectively. Univariable and multivariable logistic regression analyses after adjustment for age, gender, body mass index (BMI), traditional risk factors for coronary artery disease including smoking, hypertension, hyperlipidemia and diabetes, prior myocardial infarction, multi-vessel disease, GFR, hsCRP, left ventricular ejection fraction and use of statins were performed to detect the relationship between poor collateralization and serum levels of Lp(a) and cholesterol-containing lipids (total cholesterol, LDL-C, HDL-C and non-HDL-C). All analyses used 2-sided tests with an overall significance level of alpha = 0.05. SPSS 20.0 for Windows (SPSS, Inc., Chicago, IL, USA) was used for statistical analyses.

## Results

### Baseline characteristics

Among overall 1284 patients, poor coronary collateralization occurred in 323 diabetic (45.8%) and 182 non-diabetic patients (31.5%), respectively (P < 0.001). Both diabetic and non-diabetic patients with poor coronary collateralization were older, females and cigarette smokers in higher percentage and had more dyslipidemia but were less hypertensive than those with good collateralization (for all comparisons, P < 0.05). Biochemical tests showed hsCRP levels were more elevated but GFR was lower in patients with poor collateralization. There were no significant differences in the severity of coronary artery disease and medical treatments between the two groups (Table 1).

**Table 1:** Baseline characteristic, biochemical assessment and medication in diabetic and non-diabetic patients with poor and good collateralization

	Overall			Diabetes			Non-diabetes		
	Poor collateralization (n = 505)	Good collateralization (n = 779)	P value	Poor collateralization (n = 323)	Good collateralization (n = 383)	P value	Poor collateralization (n = 182)	Good collateralization (n = 396)	P value
Female, n (%)	137 (27.1)	120 (15.4)	< 0.001	97 (30.0)	75 (19.6)	0.001	40 (22.0)	45 (11.4)	0.001
Age	66.7 ± 10.2	62.6 ± 10.4	< 0.001	66.9 ± 10.4	63.4 ± 10.3	< 0.001	66.2 ± 10.0	61.8 ± 10.4	< 0.001
BMI, kg/m^2^	25.2 ± 3.1	25.3 ± 3.2	0.330	25.2 ± 3.0	25.5 ± 3.3	0.358	25.0 ± 3.1	25.2 ± 3.1	0.483
Hypertension, n (%)	313 (62.0)	562 (72.1)	< 0.001	202 (62.5)	285 (74.4)	0.001	111 (61.0)	277 (69.9)	0.033
Diabetes mellitus, n (%)	323 (64.0)	383 (49.2)	< 0.001	323 (100.0)	383 (100.0)	–	0 (0.0)	0 (0.0)	–
Dyslipidemia, n (%)	137 (27.1)	110 (14.1)	< 0.001	101 (31.3)	76 (19.8)	< 0.001	36 (19.8)	34 (8.6)	< 0.001
Smoking, n (%)	214 (42.4)	238 (30.6)	< 0.001	137 (42.4)	116 (30.3)	0.001	77 (42.3)	122 (30.8)	0.007
Prior MI, n (%)	150 (29.7)	187 (24.0)	0.023	91 (28.2)	87 (22.7)	0.096	59 (32.4)	100 (25.3)	0.073
Severity of CAD, n (%)			0.801			0.439			0.384
1-vessel	88 (17.4)	139 (17.8)	0.848	46 (14.2)	65 (17.0)	0.321	42 (23.1)	74 (18.7)	0.221
2-vessel	152 (30.1)	221 (28.4)	0.505	94 (29.1)	98 (25.6)	0.296	58 (31.9)	123 (31.1)	0.846
3-vessel	265 (52.5)	419 (53.8)	0.645	183 (56.7)	220 (57.4)	0.834	82 (45.1)	199 (50.3)	0.246
Multi-vessel	417 (82.6)	640 (82.2)	0.848	277 (85.6)	318 (83.0)	0.321	140 (76.9)	322 (81.3)	0.221
SBP, mmHg	136.8 ± 22.3	138.1 ± 20.6	0.283	137.6 ± 22.5	140.2 ± 19.8	0.107	135.5 ± 22.0	136.2 ± 21.1	0.699
DBP, mmHg	78.0 ± 12.9	82.3 ± 12.4	< 0.001	78.2 ± 13.2	83.2 ± 12.4	< 0.001	77.7 ± 12.2	81.6 ± 12.3	< 0.001
FBG, mmol/L	6.09 ± 2.38	5.83 ± 2.02	0.044	6.69 ± 2.74	6.61 ± 2.54	0.693	5.03 ± 0.76	5.08 ± 0.81	0.477
HbA1c, %	6.62 ± 1.27	6.46 ± 1.35	0.027	7.07 ± 1.37	7.09 ± 1.58	0.899	5.83 ± 0.36	5.85 ± 0.66	0.697
Triglyceride, mmol/L	1.74 ± 0.94	1.76 ± 1.11	0.786	1.86 ± 1.06	1.83 ± 1.18	0.715	1.52 ± 0.61	1.69 ± 1.04	0.053
Total cholesterol, mmol/L	5.00 ± 1.02	4.64 ± 1.08	< 0.001	5.10 ± 1.00	4.77 ± 1.12	< 0.001	4.82 ± 1.03	4.51 ± 1.02	0.001
LDL-C, mmol/L	3.22 ± 0.81	2.94 ± 0.83	< 0.001	3.27 ± 0.79	3.01 ± 0.88	< 0.001	3.11 ± 0.84	2.87 ± 0.77	0.001
HDL-C, mmol/L	0.98 ± 0.24	1.01 ± 0.25	0.098	0.98 ± 0.25	0.99 ± 0.24	0.304	0.99 ± 0.24	1.02 ± 0.26	0.314
Non-HDL-C, mmol/L	4.02 ± 1.04	3.64 ± 1.08	< 0.001	4.12 ± 1.02	3.78 ± 1.11	< 0.001	3.82 ± 1.04	3.50 ± 1.03	< 0.001
Lp(a), mg/dL	23.82 (12.42–44.10)	16.87 (9.28–32.2)	< 0.001	23.76 (12.97–43.48)	16.87 (9.30–31.72)	< 0.001	24.09 (12.42–45.17)	16.78 (8.56–32.34)	0.001
BUN, mmol/L	5.8 ± 1.9	5.8 ± 2.0	0.730	6.0 ± 2.0	5.9 ± 2.0	0.558	5.6 ± 1.8	5.7 ± 2.0	0.465
Serum creatinine, μmol/L	88 ± 25	84 ± 29	0.007	90 ± 28	84 ± 30	0.003	86 ± 18	85 ± 28	0.723
Uric acid, μmol/L	342 ± 85	340 ± 90	0.707	340 ± 87	330 ± 89	0.129	344 ± 81	349 ± 90	< 0.001
GFR, mL/min/1.73 m^2^	75.4 ± 17.7	83.7 ± 19.4	< 0.001	74.1 ± 18.6	83.2 ± 19.5	< 0.001	77.7 ± 15.9	84.2 ± 19.3	0.530
hsCRP, mmol/L	4.28 (1.61–7.68)	2.72 (1.12–4.85)	< 0.001	4.52 (1.74–7.89)	2.86 (1.32–5.07)	< 0.001	3.20 (1.43–6.90)	2.50 (0.86–4.51)	< 0.001
LVEF, %	57.4 ± 8.4	61 ± 8.2	< 0.001	57.0 ± 8.4	60.6 ± 7.5	< 0.001	58.1 ± 8.3	61.4 ± 8.7	< 0.001
Medication, n (%)									
Antiplatelet	374 (74.1)	552 (70.9)	0.212	236 (73.1)	274 (71.5)	0.652	138 (75.8)	278 (70.2)	0.162
ACE inhibitors/ ARBs	312 (61.8)	476 (61.1)	0.807	194 (60.1)	235 (61.4)	0.725	118 (64.8)	241 (60.9)	0.360
β-blockers	244 (48.3)	379 (48.7)	0.907	152 (47.1)	171 (44.6)	0.522	92 (50.5)	208 (52.5)	0.659
CCBs	152 (30.1)	208 (26.7)	0.185	101 (68.7)	107 (27.9)	0.333	51 (28.0)	101 (25.5)	0.591
Diuretics	61 (12.1)	81 (10.4)	0.348	39 (12.1)	42 (11.0)	0.645	22 (12.1)	39 (9.8)	0.416
Nitrates	270 (53.5)	427 (54.8)	0.636	182 (56.3)	230 (60.1)	0.320	88 (48.4)	197 (49.7)	0.755
Statins	324 (64.2)	515 (66.1)	0.473	188 (58.2)	226 (59.0)	0.829	136 (74.7)	289 (73.0)	0.659

Data are mean ± SD or number (%)

*ACE* angiotensin converting enzyme, *ARB* angiotensin receptor blocker, *BMI* body mass index, *BUN* blood urea nitrogen, *CAD* coronary artery disease, *CCB* calcium channel blocker, *DBP* diastolic blood pressure, *FBG* fasting blood glucose, *GFR* estimated glomerular filtration rate, *HbA1c* glycosylated hemoglobin A1c, *HDL-C* high-density lipoprotein cholesterol, *hsCRP* high-sensitivity C reactive protein, *LDL-C* low-density lipoprotein cholesterol, *Lp(a)* lipoprotein a, *LVEF* left ventricular ejection fraction, *MI* myocardial infarction, *SBP* systolic blood pressure

### Lp(a) and lipid profile

In diabetic and non-diabetic settings, patients with poor coronary collateralization had higher serum levels of Lp(a), total cholesterol, LDL-C, and non-HDL-C compared to those with good collateralization (P ≤ 0.001), but HDL-C and triglyceride levels were similar (Table 1). In diabetic patients, Lp(a) correlated with total cholesterol (adjusted r = 0.080, P = 0.035), LDL-C (adjusted r = 0.076, P = 0.045), non-HDL-C (adjusted r = 0.090, P = 0.017) and triglyceride (adjusted r = − 0.113, P = 0.003) but was not related to HDL-C (P = 0.231) after adjustment for gender, age, BMI, risk factors for coronary artery disease (hypertension, dyslipidemia, smoking), prior myocardial infarction, multi-vessel disease, renal function, log-transferred hsCRP and left ventricular ejection fraction. In non-diabetic patients, such a significant correlation was not found (P = 0.053–0.087). After adjustment for these potential risk factors, tertiles of Lp(a) (adjusted OR = 1.366, 95% CI 1.108–1.684, P = 0.003 and adjusted OR = 1.432, 95% CI 1.119–1.831, P = 0.004), total cholesterol (adjusted OR = 1.814, 95% CI 1.393–2.361, P < 0.001 and adjusted OR = 1.820, 95% CI 1.358–2.440, P < 0.001), LDL-C (adjusted OR = 1.830, 95% CI 1.407–2.381, P < 0.001 and adjusted OR = 1.699, 95% CI 1.270–2.274, P < 0.001) and non-HDL-C (adjusted OR = 1.810, 95% CI 1.386–2.364, P < 0.001 and adjusted OR = 1.912, 95% CI 1.407–2.597, P < 0.001) remained independent determinants for poor collateralization in diabetic and non-diabetic patients (Table 2). Three lipid measurements (total cholesterol, LDL-C and non-HDL-C) with significant difference between poor and good collaterals (Table 1) were chosen for further adjustment, and Lp(a) was still independently associated with collateralization in diabetics and non-diabetics (Additional file 1: Table S1). The result patterns were similar in additional analysis with the quartiles of Lp(a), total cholesterol, LDL-C, non-HDL-C, HDL-C and triglyceride (Additional file 2: Table S2).

**Table 2:** Impact of lipid profile on poor collateralization in patients with and without diabetes

	Tertiles of lipid profile		Overall (n = 1284)			Diabetes (n = 706)			Non-diabetes (n = 578)		
	Range	n	Poor/good	Adjusted OR (95% CI)^a^	P value	Poor/good	Adjusted OR (95% CI)^a^	P value	Poor/good	Adjusted OR (95% CI)^a^	P value
Lp(a)	Per tertile, mg/dL			1.394 (1.192–1.629)	< 0.001*		1.366 (1.108–1.684)	0.003*		1.432 (1.119–1.831)	0.004*
	T1 < 12.66	426	128/298	1	–	80/147	1	–	48/151	1	–
	12.66 ≤ T2 < 30.23	449	176/273	1.170 (0.861–1.591)	0.316	117/134	1.282 (0.854–1.926)	0.231	59/139	1.040 (0.640–1.688)	0.875
	T3 ≥ 30.23	409	201/208	1.929 (1.413–2.633)	< 0.001	126/102	1.863 (1.227–2.830)	0.004	75/106	2.010 (1.236–3.269)	0.005
Total cholesterol	Per tertile, mmol/L			1.791 (1.475–2.174)	< 0.001*		1.814 (1.393–2.361)	< 0.001*		1.820 (1.358–2.440)	< 0.001*
	T1 < 4.26	420	123/297	1	–	66/129	1	–	57/168	1	–
	4.26 ≤ T2 < 5.35	441	170/271	1.612 (1.168–2.224)	0.004	109/129	1.993 (1.251–3.173)	0.004	61/142	1.357 (0.854–2.158)	0.196
	T3 ≥ 5.35	423	212/211	3.221 (2.186–4.747)	< 0.001	148/125	3.337 (1.962–5.674)	< 0.001	64/86	3.663 (2.010–6.675)	< 0.001
LDL-C	Per tertile, mmol/L			1.748 (1.441–2.122)	< 0.001*		1.830 (1.407–2.381)	< 0.001*		1.699 (1.270–2.274)	< 0.001*
	T1 < 2.58	401	115/286	1	–	61/138	1	–	54/148	1	–
	2.58 ≤ T2 < 3.36	431	171/260	1.620 (1.164–2.255)	0.004	116/115	2.426 (1.518–3.879)	< 0.001	55/145	1.052 (0.648–1.709)	0.837
	T3 ≥ 3.36	452	219/233	3.048 (2.070–4.488)	< 0.001	146/130	3.509 (2.064–5.964)	< 0.001	73/103	3.092 (1.724–5.548)	< 0.001
Non-HDL-C	Per tertile, mmol/L			1.828 (1.498–2.230)	< 0.001*		1.810 (1.386–2.364)	< 0.001*		1.912 (1.407–2.597)	< 0.001*
	T1 < 3.30	436	131/305	1	–	71/131	1	–	60/174	1	–
	3.30 ≤ T2 < 4.38	441	167/274	1.540 (1.122–2.113)	0.008	104/133	1.683 (1.073–2.639)	0.023	63/141	1.407 (0.891–2.222)	0.143
	T3 ≥ 4.38	407	207/200	3.422 (2.293–5.108)	< 0.001	148/119	3.263 (1.911–5.571)	< 0.001	59/81	4.260 (2.245–8.086)	< 0.001
HDL-C	Per tertile, mmol/L			0.907 (0.775–1.060)	0.220*		0.846 (0.686–1.045)	0.121*		0.994 (0.780–1.267)	0.960*
	T1 < 0.86	388	172/216	1	–	117/112	1	–	55/104	1	–
	0.86 ≤ T2 < 1.04	437	161/276	0.809 (0.596–1.098)	0.174	101/133	0.808 (0.540–1.210)	0.302	60/143	0.834 (0.514–1.354)	0.463
	T3 ≥ 1.04	459	172/287	0.820 (0.600–1.121)	0.214	105/138	0.717 (0.471–1.093)	0.122	67/149	0.975 (0.602–1.579)	0.918
Triglyceride	Per tertile, mmol/L			1.075 (0.914–1.266)	0.383*		1.092 (0.880–1.352)	0.428*		1.055 (0.816–1.365)	0.683*
	T1 < 1.28	436	158/278	1	–	96/122	1	–	62/156	1	–
	1.28 ≤ T2 < 1.84	448	175/273	1.109 (0.822–1.497)	0.498	99/135	0.947 (0.625–1.434)	0.796	76/138	1.311 (0.840–2.046)	0.232
	T3 ≥ 1.84	400	172/228	1.155 (0.833–1.600)	0.387	128/126	1.183 (0.771–1.817)	0.442	44/102	1.072 (0.633–1.814)	0.796

*CI* confidence interval, *HDL-C* high-density lipoprotein cholesterol, *LDL-C* low-density lipoprotein cholesterol, *Lp(a)* lipoprotein a, *OR* odds ratio

* P for trend for tertiles of lipid profile

^a^Multiple adjustment for gender, age, body mass index, hypertension, diabetes, dyslipidemia, smoking, prior myocardial infarction, multi-vessel disease, glomerular filtration rate, log-transferred high-sensitivity C reactive protein and left ventricular ejection fraction

**Fig. 2:**
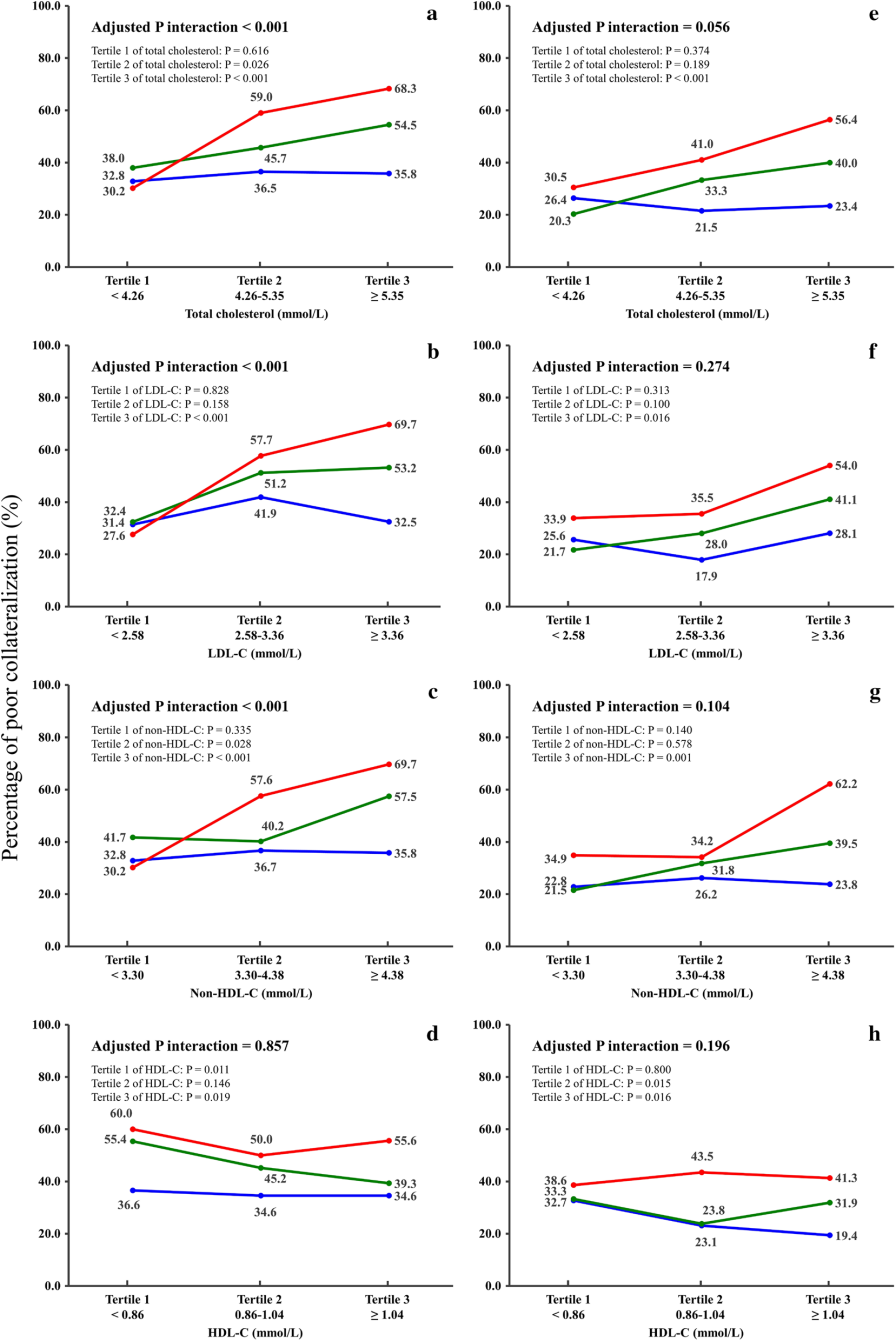
Percentage of poor coronary collateralization in relation to interaction between Lp(a) and total cholesterol, LDL-C, non-HDL-C or HDL-C in diabetics (**a**–**d**) and non-diabetics (**e**–**h**). Values are percentage of poor collateralization according to tertile distribution of Lp(a) (blue, green and red line for tertile 1, 2 and 3, respectively). P values for poor collateralization for each tertile of total cholesterol, LDL-C, non-HDL-C and HDL-C are given

For patients with diabetes, there was a significant interaction between Lp(a) and total cholesterol, LDL-C or non-HDL-C in relation to poor coronary collateralization (all adjusted P interaction < 0.001). At high tertile of total cholesterol (≥ 5.35 mmol/L), LDL-C (≥ 3.36 mmol/L) and non-HDL-C (≥ 4.38 mmol/L), patients with high tertile of Lp(a) (≥ 30.23 mg/dL) had a significantly increased risk of poor collateralization compared with those with low tertile of Lp(a) (< 12.66 mg/dL) (adjusted OR = 4.300, 95% CI 2.095–8.826, adjusted OR = 3.970, 95% CI 1.918–8.216 and adjusted OR = 4.386, 95% CI 2.115–9.094, respectively, all P < 0.001) (Fig. 2). Furthermore, the additional inclusion of interaction of Lp(a) with total cholesterol, LDL-C and non-HDL-C provided better risk prediction of poor coronary collateralization with a significantly improved goodness-of-fit and predictive performance with an increase of Nagelkerke R^2^ of 3.4% (< 0.001) for total cholesterol, 3.7% (P < 0.001) for LDL-C and 3.4% (P < 0.001) for non-HDL-C, respectively, and C statistic of 0.019 (95% CI 0.003–0.034, P = 0.016) for total cholesterol, 0.019 (95% CI 0.003–0.034, P = 0.016) for LDL-C and 0.018 (95% CI 0.003–0.033, P = 0.020) for non-HDL-C, respectively. However, there was no interaction of Lp(a) with HDL-C and triglyceride on coronary collateralization (adjusted P interaction = 0.857 and 0.941, respectively).

For non-diabetic patients, no such interactions between Lp(a) and cholesterol-containing lipids and triglyceride were observed (Fig. 2).

## Discussion

The results of this large cohort study support the hypothesis that in patients with stable coronary artery disease and chronic total occlusion, increased Lp(a) confers greater risk for poor coronary collateralization when total cholesterol, LDL-C or non-HDL-C are elevated especially in patients with type 2 diabetes.

### Relation between Lp(a) and coronary collateralization in diabetes

It is suggested that there is an inverse association between Lp(a) concentration and risk of type 2 diabetes, with a higher risk for type 2 diabetes at low Lp(a) concentrations—approximately < 7 mg/dL [31]. Concerning type 1 diabetes, no different levels of Lp(a) were found between patients with any degree of coronary stenosis and those without coronary disease [32]. The present finding on an inverse association between elevated Lp(a) levels and poor coronary collateral formation is consistent with previous reports examining the same phenomenon [14, 15]. Nevertheless, our study population was unique as all patients had stable coronary artery disease and chronic total occlusion. This study is also the first to investigate the relation of Lp(a) and its interactions with a broad spectrum of cholesterol-containing lipids including total cholesterol, LDL-C, HDL-C, and non-HDL-C on coronary collateralization in a large number of patients with type 2 diabetes. Our results showed that the prevalence of elevated Lp(a) levels was higher in patients with poor coronary collateralization as compared to those with good collateralization, and notably, tertiles of Lp(a) remained an independent determinant for poor collateralization even after adjustment for various confounding factors including cholesterol-containing lipid profiles. These observations support a notion that Lp(a) could be a biomarker of coronary collateral circulation in diabetic patients with stable coronary artery disease and chronic total occlusion. Although its physiological function is still not completely elucidated, Lp(a) is known to be highly concentrated in the arterial wall, carries cholesterol and binds atherosclerogenic oxidized phospholipids, which attracts inflammatory cells to vessel walls and leads to smooth muscle cell proliferation, consequently, contributing to the process of atherosclerosis [16, 19]. Several studies have shown that high levels of Lp(a) adversely affect collateral vessel growth by inducing endothelial cell dysfunction through various mechanisms [33]. Aras et al found a strong negative correlation between serum Lp(a) levels and vascular endothelial growth factor concentration in patients with chronic total coronary occlusion [14]. High levels of Lp(a) attenuate synthesis and/or release of vascular endothelial growth factor and decrease production of endothelium-derived nitric oxide, leading to impaired angiogenesis [15]. Using a Lp(a) transgenic mouse hindlimb ischemia model, Morishita et al revealed that a high serum Lp(a) concentration abolished collateral formation by inhibition of transforming growth factor-β activity, suggesting that Lp(a) might also decrease arteriogenesis [34].

### Lp(a) interactions with LDL-C and non-HDL-C on coronary collateralization in diabetes

The major finding of this study is that the adverse effect of Lp(a) on coronary collateral development was aggravated by the presence of a high level of LDL-C or non-HDL in patients with diabetes. Our results showed that Lp(a) was significantly correlated with LDL-C or non-HDL-C, and individuals with high Lp(a) were more likely to have LDL-C > 3.36 mmol/L or non-HDL-C > 4.38 mmol/L, confirming the physiological link between Lp(a) and LDL-C or non-HDL-C. Furthermore, there was a synergistic effect of Lp(a) and LDL-C or non-HDL-C on collateral formation in patients with diabetes. In high tertile of LDL-C or non-HDL-C, diabetic patients with high tertile of Lp(a) had an approximately fourfold increased risk of poor coronary collateralization compared with those with low tertile of Lp(a). These observations suggest that Lp(a) might exert a more pronounced detrimental effect on coronary collateral formation in a high-risk lipid profile environment.

Although certain studies have not shown any significant difference in the prevalence of elevated LDL-C in patients with diabetes compared with non-diabetic counterparts [35], total LDL-C may be a misleading measurement in diabetes. This may be in part due to a significant shift to small dense LDL-C in diabetic dyslipidemia, which is associated with greater cardiovascular disease risk. In fact, the same level of LDL-C can be associated with greater serum levels of apo B and hence, more low-density lipoprotein particles. Whether the adverse effects of Lp(a) interactions with LDL-C on coronary collateralization in patients with diabetes is due to these changes remains unknown and requires further investigations.

Non-HDL-C encompasses all of the atherogenic apoB-containing lipoproteins (LDL-C, very low-density lipoprotein cholesterol, intermediate-density lipoprotein cholesterol) [18]. Achieved non-HDLC levels reflect the full burden of cholesterol transported in atherogenic lipoproteins, and seem to be more closely associated with coronary atheroma progression than LDL-C even among statin-treated individuals [36]. Recently, both the International Atherosclerosis Society and National Lipid Association have flagged non-HDL-C as the major form of atherogenic cholesterol and recommended using this parameter as the marker for the primary and secondary prevention of atherosclerotic cardiovascular disease [20, 37, 38]. The mechanism of elevated non-HDL-C on poor coronary collateralization is likely to be complex. Lipid profile impairment, especially hypercholesterolemia and high levels of LDL-C and non-HDL, is an established risk factor that induces endothelial cell dysfunction and impairs coronary collateral vessel growth [18]. Alvim et al found that hypercholesterolemia and high non-HDL-C levels were associated with increased arterial stiffness characterized by elevated systolic and pulse blood pressures and reduced diastolic blood pressure [39]. Baykan et al observed that increased arterial stiffness could cause decreased coronary perfusion, reduces shear stress, arteriogenesis, and thus collateral formation [40]. Our data were partially corroborated by the results of several population studies demonstrating that non-HDL-C is a better marker of cardiovascular disease risk than LDL-C alone [36, 41].

### Relation between HDL-C and coronary collateralization in diabetes

It has been well recognized that HDL-C has anti-atherogenic, anti-inflammatory, and anti-oxidant properties [42]. Sumi et al. observed that reconstituted HDL-C directly stimulates endothelial progenitor cell differentiation via phosphatidylinositol 3-kinase/Akt pathway and enhances ischemia-induced angiogenesis [43]. The relation between serum HDL-C and coronary collateral formation remains controversial. Kadi et al found that in patients with stable coronary artery disease there was a positive relationship between HDL-C and angiographic collateral score, and low HDL-C is an independent determinant of poor coronary collateralization [44]. Recently, Hsu et al reported that the extent of coronary artery disease severity but not HDL-C level was the most powerful predictor of coronary collateral formation in 501 Chinese patients with stable coronary artery disease [45]. In the present study, we did not find an association between serum HDL-C level and coronary collateral score. This finding is in line with our previous observations that HDL-C functionality rather than quantity alone may reflect its overall effect [46, 47]. However, the detailed mechanism still needs further investigation.

### Clinical implications

The present study demonstrates the robust associations between Lp(a) interactions with cholesterol-containing lipids and coronary collateral formation in patients with stable coronary artery disease and chronic total occlusion, which were not linear and limited to highest Lp(a) and LDL-C or non-HDL-C tertile. These observations emphasize the potential importance of LDL-C and non-HDL-C in pointing to patients at increased risk for Lp(a)-mediated disease, as well as preventative strategies to mitigate the risk conferred by elevated Lp(a) levels (e.g., LDL-C or non-HDL-C lowering) especially for patients with diabetes. First, our study substantiates the concept that LDL-C or non-HDL reduction with statin therapy remains the mainstay of pharmacotherapy for dyslipidemia and the percentage reduction in LDL-C lowering is strongly correlated with reduction in atherosclerotic cardiovascular disease risk and events [18–20]. Recent trials using cholesterol absorption blocker ezetimibe in combination with statins [48] and proprotein convertase subtilisin/kexin type 9 (PCSK-9) inhibitors [49] clearly show that greater LDL-C reduction on top of statin therapy provides added benefits, without attenuation in cardiovascular disease benefit in patients starting treatment with lower LDL-C. In addition, dipeptidyl peptidase-4 (DDP4) inhibitor anagliptin has been shown to have inhibitory effects on hepatic cholesterol synthesis and reduce LDL-C by 9.5 mg/dL over 12 weeks,regardless of the use of statins [50]. Second, our study highlights the need for Lp(a) lowering therapy [51–53]. Ezetimibe treatment either alone or in combination with a statin does not affect serum Lp(a) concentration [54] and fibrates have a significantly greater effect in reducing serum Lp(a) than statins. Addition of fibrates to statins can enhance the Lp(a)-lowering effect of statins [55]. Nicotinic acid has been shown to decrease Lp(a) levels by 15–30% and reduce cardiovascular events [56]. However, even slower release niacin is usually poorly tolerated because of gastrointestinal side effects, making it less popular with patients [57]. PCSK-9 inhibitors are promising as these agents could lower Lp(a) with various degree among patients [49, 51, 56]. Likewise, apo(a) antisense oligonucleotides is a new treatment option for lowering elevated Lp(a). Clinical trials of Lp(a) reduction using antisense oligonucleotides that lower apo(a) levels by 90% are underway. For some patients with diabetes and increased Lp(a), such therapies may enter the clinic soon [58, 59]. The long-term efficacy of lipoprotein apheresis on morbidity in patients with elevated Lp(a) and chronic ischemic heart disease on maximally tolerated lipid-lowering therapy is well documented [60].

### Study limitations

We recognize that there are several limitations in our study. First, the study is cross-sectional for the point of coronary collateral investigation, thereby allowing us to detect association, not to formulate causal link. In addition, the correlation of Lp(a) with total cholesterol and LDL-C was statistically significant but very weak. It remains uncertain whether we will be able to use the recent data for any recommendations, as the Lp(a) tertiles are completely not specific. Second, the presence and degree of collaterals were evaluated according to the Rentrop scoring system. Although this angiographic assessment of coronary collaterals is easily to be incorporated into the routine clinical practice, coronary collaterals may be more accurately assessed by collateral flow index with simultaneous measurement of aortic pressure and the distal pressure within the occluded segment of the culprit coronary artery [61].

## Conclusions

In patients with stable coronary artery disease and chronic total occlusion, poor coronary collateralization seems tightly linked to Lp(a) interactions with total cholesterol, LDL-C, and non-HDL-C. Individuals with high level of Lp(a) (> 30.23 mg/dL) and total cholesterol (> 5.35 mmol/L), LDL-C (> 3.36 mmol/L) or non-HDL-C (> 4.38 mmol/L) may warrant aggressive lipid lowing therapy especially for those with type 2 diabetes.

## Additional files

**Additional file 1 :Table S1.** Logistic regression analyses for poor collateralization in patients with and without diabetes.

**Additional file 2: Table S2.** Impact of lipid profile on poor collateralization in patients with and without diabetes.

## Data Availability

Data generated or analyzed during this study are included in this published article.

## Abbreviations

apo: apolipoprotein
BMI: body mass index
CKD-EPI: chronic kidney disease epidemiology collaboration
GFR: glomerular filtration rate
HbA1c: glycated hemoglobin
HDL-C: high-density lipoprotein cholesterol
hsCRP: high-sensitivity C-reactive protein
LDL-C: low-density lipoprotein cholesterol
Lp(a): lipoprotein (a)
PCSK9: proprotein convertase subtilisin/kexin type 9
SD: standard deviation

## References

[1] Seiler C, Stoller M, Pitt B, Meier P (2013). The human coronary collateral circulation: development and clinical importance. Eur Heart J.

[2] Zimarino M, D’Andreamatteo M, Waksman R, Epstein SE, De Caterina R (2014). The dynamics of the coronary collateral circulation. Nat Rev Cardiol.

[3] Jamaiyar A, Juguilon C, Dong F, Cumpston D, Enrick M, Chilian WM, Yin L (2019). Cardioprotection during ischemia by coronary collateral growth. Am J Physiol Heart Circ Physiol.

[4] Werner GS (2014). The role of coronary collaterals in chronic total occlusion. Curr Cardiol Rev.

[5] Shen Y, Ding FH, Dai Y, Wang XQ, Zhang RY, Lu L, Shen WF (2018). Reduced coronary collateralization in type 2 diabetic patients with chronic total occlusion. Cardiovasc Diabetol.

[6] Shen Y, Lu L, Ding FH, Sun Z, Sun Z, Zhang RY, Zhang Q, Yang ZK, Hu J, Chen QJ (2013). Association of increased serum glycated albumin levels with low coronary collateralization in type 2 diabetic patients with stable angina and chronic total occlusion. Cardiovasc Diabetol.

[7] Werner GS, Richartz BM, Heinke S, Ferrari M, Figulla HR (2003). Impaired acute collateral recruitment as a possible mechanism for increased cardiac adverse events in patients with diabetes mellitus. Eur Heart J.

[8] Shen Y, Ding FH, Zhang RY, Zhang Q, Lu L, Shen WF (2016). Association of serum mimecan with angiographic coronary collateralization in patients with stable coronary artery and chronic total occlusion. Atherosclerosis.

[9] Shen Y, Lu L, Liu ZH, Wu F, Zhu JZ, Sun Z, Zhang RY, Zhang Q, Hu J, Chen QJ (2014). Increased serum level of CTRP1 is associated with low coronary collateralization in stable angina patients with chronic total occlusion. Int J Cardiol.

[10] Nordestgaard BG, Chapman MJ, Ray K, Boren J, Andreotti F, Watts GF, Ginsberg H, Amarenco P, Catapano A, Descamps OS, European Atherosclerosis Society Consensus Panel (2010). Lipoprotein(a) as a cardiovascular risk factor: current status. Eur Heart J.

[11] Kamstrup PR, Tybjaerg-Hansen A, Steffensen R, Nordestgaard BG (2009). Genetically elevated lipoprotein(a) and increased risk of myocardial infarction. JAMA.

[12] Mitroshkin M, Ezhov M, Safarova M (2016). Lipoprotein(a) level as a predictor of long-term cardiovascular outcomes after percutaneous coronary interventions. Atherosclerosis.

[13] Afshar M, Pilote L, Dufresne L, Engert JC, Thanassoulis G (2016). Lipoprotein(a) interactions with low-density lipoprotein cholesterol and other cardiovascular risk factors in premature acute coronary syndrome (ACS). J Am Heart Assoc.

[14] Aras D, Geyik B, Topaloglu S, Ergun K, Ayaz S, Maden O, Yildiz A, Balci M, Ozeke O, Korkmaz S (2006). Serum level of lipoprotein (a) is inversely associated with the development of coronary collateral circulation. Coron Artery Dis.

[15] Fan Y, Hu JS, Guo F, Lu ZB, Xia H (2017). Lipoprotein(a) as a predictor of poor collateral circulation in patients with chronic stable coronary heart disease. Braz J Med Biol Res.

[16] Wu HD, Berglund L, Dimayuga C, Jones J, Sciacca RR, Di Tulio MR (2004). High lipoprotein (a) levels and small apolipoprolein (a) sizes are associated with endothelial dysfunction in a multi-ethnic cohort. J Am Coll Cardiol.

[17] Duan J, Murohara T, Ikeda H, Katoh A, Shintani S, Sasaki K (2000). Hypercholesterolemia inhibits angiogenesis in response to hindlimb ischemia. Circulation.

[18] Puri R, Nissen SE, Shao M, Elshazly MB, Kataoka Y, Kapadia SR, Tuzcu EM, Nicholls SJ (2016). Non-HDL cholesterol and triglycerides: implications for coronary atheroma progression and clinical events. Arterioscler Thromb Vasc Biol.

[19] Ray KK, Ginsberg HN, Davidson MH, Pordy R, Bessac L, Minini P, Eckel RH, Cannon CP (2016). Reductions in atherogenic lipids and major cardiovascular events: a pooled analysis of 10 ODYSSEY trials comparing alirocumab with control. Circulation.

[20] Joint committee for guideline revision (2018). 2016 Chinese guidelines for the management of dyslipidemia in adults. J Geriatr Cardiol.

[21] Kennedy MW, Fabris E, Suryapranata H, Kedhi E (2017). Is ischemia the only factor predicting cardiovascular outcomes in all diabetes mellitus patients?. Cardiovasc Diabetol.

[22] Du R, Zhang RY, Lu L, Shen Y, Pu LJ, Zhu ZB, Zhang Q, Hu J, Yang ZK, Ding FH (2018). Increased glycated albumin and decreased esRAGE levels in serum are related to negative coronary artery remodeling in patients with type 2 diabetes: an intravascular ultrasound study. Cardiovasc Diabetol.

[23] Hinkel R, Howe A, Renner S, Ng J, Lee S, Klett K, Kaczmarek V, Moretti A, Laugwitz KL, Skroblin P (2017). Diabetes mellitus-induced microvascular destabilization in the myocardium. J Am Coll Cardiol.

[24] American Diabetes Association (2014). Standards of medical care in diabetes—2014. Diabetes Care.

[25] Miedema MD, Lopez FL, Blaha MJ, Virani SS, Coresh J, Ballantyne CM, Folsom AR (2015). Implications of the eighth joint national committee guidelines for the management of high blood pressure for aging adults: atherosclerosis risk in communities study. Hypertension.

[26] Executive Summary of the Third Report of The National Cholesterol Education Program (NCEP) (2001). Expert panel on detection, evaluation, and treatment of high blood cholesterol in adults (Adult Treatment Panel III). JAMA.

[27] Fraker TD, Fihn SD, Gibbons RJ, Abrams J, Chatterjee K, Daley J, Deedwania PC, Douglas JS, Ferguson TB, Gardin JM (2007). 2007 chronic angina focused update of the ACC/AHA 2002 guidelines for the management of patients with chronic stable angina: a report of the American College of Cardiology/American Heart Association Task Force on Practice Guidelines Writing Group to develop the focused update of the 2002 guidelines for the management of patients with chronic stable angina. J Am Coll Cardiol.

[28] Levy AS, Stevens LA, Schmid CH, Zhang YL, Castro AF, Feldman HI, Kusek JW, Eggers P, Van Lente F, Greene T, CKD-EPI (Chronic Kidney Disease Epidemiology Collaboration), CKD-EPI (Chronic Kidney Disease Epidemiology Collaboration) (2009). A new equation to estimate glomerular filtration rate. Ann Intern Med.

[29] Ellis SG, Vandormael MG, Cowley MJ, DiSciascio G, Deligonul U, Topol EJ, Bulle TM (1990). Coronary morphologic and clinical determinants of procedural outcome with angioplasty for multivessel coronary disease: implications for patient selection (Multivessel Angioplasty Prognosis Study Group). Circulation.

[30] Rentrop KP, Cohen M, Blanke H, Phillips RA (1985). Changes in collateral channel filling immediately after controlled coronary artery occlusion by an angioplasty balloon in human subjects. J Am Coll Cardiol.

[31] Paige (2017). Lipoprotein(a) and incident type-2 diabetes: results from the prospective Bruneck study and a meta-analysis of published literature. Cardiovasc Diabetol.

[32] Colom (2018). Associations between epicardial adipose tissue, subclinical atherosclerosis and high-density lipoprotein composition in type 1 diabetes. Cardiovasc Diabetol.

[33] Banach M, Aronow WS, Serban C, Sahabkar A, Rysz J, Voroneanu L, Covic A (2015). Lipid, blood pressure and kidney update 2014. Pharmacol Res.

[34] Morishita R, Sakaki M, Yamamoto K, Iguchi S, Aoki M, Yamasaki K (2002). Impairment of collateral formation in lipoprotein(a) transgenic mice: therapeutic angiogenesis induced by human hepatocyte growth factor gene. Circulation.

[35] Nelson AJ, Rochelau SK, Nicholis SJ (2018). Managing dyslipidemia in type 2 diabetes. Endocrinol Metab Clin N Am.

[36] Ito T, Arima H, Fujiyoshi A, Miura K, Takashima N, Ohkubo T, Kadota A, Hayakawa T, Kita Y, Miyagawa N, NIPPON DATA90 Research Group (2016). Relationship between non-high-density lipoprotein cholesterol and the long-term mortality of cardiovascular diseases: NIPPON DATA 90. Int J Cardiol.

[37] Jacobson TA, Ito MK, Maki KC, Orringer CE, Bays HE, Jones PH, McKenney JM, Grundy SM, Gill EA, Wild RA (2015). National lipid association recommendations for patient-centered management of dyslipidemia: part 1—full report. J Clin Lipidol.

[38] Grundy SM, Expert Dyslipidemia Panel (2013). An international atherosclerosis society position paper: global recommendations for the management of dyslipidemia. J Clin Lipidol.

[39] Alvim RO, Mourao-Junior CA, Magalha GL, de Oliveira CM, Krieger JE, Mill JG (2017). Non-HDL cholesterol is a good predictor of the risk of increased arterial stiffness in postmenopausal women in an urban Brazilian population. Clinics.

[40] Baykan AO, Gur M, Acele A, Seker T, Quisi A, Yildirin A (2015). Coronary collateral development and arterial stiffness in patients with chronic coronary total occlusion. Scan Cardiovasc J.

[41] Zhang N, Hu X, Zhang Q, Bai P, Cai M, Zeng TS, Zhang JY, Tian SH, Min J, Huang HT (2018). Non-high-density lipoprotein cholesterol: high-density lipoprotein cholesterol ratio is an independent risk factor for diabetes mellitus: results from a population-based cohort study. J Diabetes.

[42] Sun JT, Shen Y, Lü AK, Lu L, Shen WF (2013). Glycation of high-density lipoprotein in type 2 diabetes mellitus. Chin Med J.

[43] Sumi M, Sata M, Miura S, Rye KA, Toya N, Kanaoka Y, Yanaga K, Ohki T, Saku K, Nagai R (2007). Reconstituted high-density lipoprotein stimulates differentiation of endothelial progenitor cells and enhances ischemia induced angiogenesis. Arterioscler Thromb Vasc Biol.

[44] Kadi H, Ozyurt H, Ceyhan K, Koc F, Celik A, Burucu T (2012). The relationship between high-density lipoprotein cholesterol and coronary collateral circulation in patients with coronary artery disease. J Investig Med.

[45] Hsu PC, Su HM, Juo SH, Yen HW, Voon WC, Lai WT, Sheu SH, Lin TH (2013). Influence of high-density lipoprotein cholesterol on coronary collateral formation in a population with significant coronary artery disease. BMC Res Notes.

[46] Shen Y, Ding FH, Sun JT, Pu LJ, Zhang RY, Zhang Q, Chen QJ, Lu L, Shen WF (2015). Association of elevated apo A-I glycation and reduced paraoxonase 1, 3 activity, and their interaction with angiographic severity of coronary artery disease in patients with type 2 diabetes. Cardiovasc Diabetol.

[47] Dai Y, Shen Y, Li QR, Ding FH, Wang XQ, Liu HJ, Yan XX, Sun JT, Wang LJ, Yang K (2017). Association of apolipoprotein A-IV glycation with CAD in T2DM patients: glycated apolipoprotein A-IV induces atherogenesis. J Am Coll Cardiol.

[48] Giugliano RP, Cannon CP, Blazing MA, Nicolau JC, Corbalán R, Špinar J, Park JG, White JA, Bohula EA, Braunwald E, IMPROVE-IT (Improved Reduction of Outcomes: Vytorin Efficacy International Trial) Investigators (2018). Benefit of adding ezetimibe to statin therapy on cardiovascular outcomes and safety in patients with versus without diabetes mellitus from IMPROVE-IT (Improved Reduction of Outcomes: Vytorin Efficacy International Trial). Circulation.

[49] Sabatine MS, Giugliano RP, Keech AC, Honarpour N, Wiviott SD, Murphy SA, Kuder JF, Wang H, Liu T, Wasserman SM, FOURIER Steering Committee and Investigators (2017). Evolocumab and clinical outcomes in patients with cardiovascular disease. N Engl J Med.

[50] Ueda S, Shimabukuro M, Arasaki O, Node K, Nomiyama T, Morimoto T (2018). Effects of anagliptin and sitagliptin on low-density lipoprotein cholesterol in type 2 diabetic patients with dyslipidemia and cardiovascular risk: rationale and study design of REASON Trial. Cardiovasc Drugs Ther.

[51] Ferretti G, Bacchetti T, Johnston TP, Banach M, Pirro M, Sahebkar A (2018). Lipoprotein(a): a missing culprit in the management of athero-thrombosis?. J Cell Physiol.

[52] Soran H, Adam S, Mohammad JB, Ho JH, Schofield JD, Kwok S, Siahmansur T, Liu Y, Syed AA, Dhage SS (2018). Hypercholesterolaemia—practical information for non-specialists. Arch Med Sci.

[53] Banach M, Jankowski P, Jóźwiak J, Cybulska B, Windak A, Guzik T, Mamcarz A, Broncel M, Tomasik T, Rysz J (2017). PoLA/CFPiP/PCS guidelines for the management of dyslipidaemias for family physicians 2016. Arch Med Sci.

[54] Sahebkar A, Simental-Mendía LE, Pirro M, Banach M, Watts GF, Sirotri C, Al-Rasadi K, Atkin SL (2018). Impact of ezetimibe on plasma lipoprotein(a) concentrations as monotherapy or in combination with statins: a systematic review and meta-analysis of randomized controlled trials. Sci Rep.

[55] Sahebkar A, Simental-Mendía LE, Watts GF, Serban MC, Banach M, Lipid and Blood Pressure Meta-analysis Collaboration (LBPMC) Group (2017). Comparison of the effects of fibrates versus statins on plasma lipoprotein(a) concentrations: a systematic review and meta-analysis of head-to-head randomized controlled trials. BMC Med.

[56] Khera AV, Oamar A, Reily MP, Dunbar RL, Rader DJ (2015). Effects of niacin, statin, and fenofibrate on circulating proprotein convertase subtilisin/kexin type 9 levels in patients with dyslipidemia. Am J Cardiol.

[57] Landray MJ, Haynes R, Hopewell JC, Parish S, Aung T, Tomson J, Wallendszus K, Craig M, Jiang L, Collins R (2014). Effects of extended-release niacin with laropiprant in high-risk patients. N Engl J Med.

[58] Graham MJ, Viney N, Crooke RM, Tsimikas S (2016). Antisense inhibitor of apolipoprotein (a) to lower plasma lipoprotein (a) levels in human. J Lipid Res.

[59] Schreml J, Gouni-Berthold I (2017). Apolipoprotein (a) antisense oligonucleotides: a new treatment option for powering elevated lipoprotein (a)?. Curr Pharm Des.

[60] Bigazzi (2018). Reduced incidence of cardiovascular events in hyper-Lp(a) patients on lipoprotein apheresis The G.I.L.A. (Gruppo Interdisciplinare Aferesi Lipoproteica) pilot study. Transfus Apher Sci.

[61] Shen Y, Yang ZK, Hu J, Wang XQ, Dai Y, Zhang S, Zhang RY, Lu L, Ding FH, Shen WF (2018). Donor artery stenosis interactions with diastolic blood pressure on coronary collateral flow in type 2 diabetic patients with chronic total occlusion. Cardiovasc Diabetol.

