# Supplementary material for: Lipoprotein (a) interactions with cholesterol-containing lipids on angiographic coronary collateralization in type 2 diabetic patients with chronic total occlusion
Source: Cardiovasc Diabetol. 2019 Jun 24;18:82. doi: 10.1186/s12933-019-0888-z (PMC6589890; doi:10.1186/s12933-019-0888-z)
Supplement: Supplementary file 1 — Additional file 1 :Table S1. Logistic regression analyses for poor collateralization in patients with and without diabetes. [file 12933_2019_888_MOESM1_ESM.docx]

Table S1. Logistic regression analyses for poor collateralization in patients with and without diabetes.

|  | Overall (n = 1284) | |  | Diabetes (n = 706) | |  | Non-diabetes (n = 578) | |
| --- | --- | --- | --- | --- | --- | --- | --- | --- |
|  | Adjusted OR (95% CI) | P value |  | Adjusted OR (95% CI) | P value |  | Adjusted OR (95% CI) | P value |
| Female | 1.916 (1.368 ~ 2.681) | < 0.001 |  | 2.004 (1.304 ~ 3.077) | 0.002 |  | 1.848 (1.053 ~ 3.247) | 0.032 |
| Age | 1.028 (1.013 ~ 1.044) | < 0.001 |  | 1.020 (1.000 ~ 1.041) | 0.054 |  | 1.044 (1.019 ~ 1.069) | 0.001 |
| Body mass index | 1.015 (0.973 ~ 1.058) | 0.492 |  | 1.020 (0.965 ~ 1.078) | 0.480 |  | 1.017 (0.952 ~ 1.087) | 0.618 |
| Hypertension | 0.654 (0.498 ~ 0.858) | 0.002 |  | 0.617 (0.427 ~ 0.892) | 0.010 |  | 0.696 (0.459 ~ 1.055) | 0.088 |
| Diabetes | 1.490 (1.145 ~ 1.938) | 0.003 |  |  |  |  |  |  |
| History of dyslipidemia | 1.505 (1.090 ~ 2.078) | 0.013 |  | 1.392 (0.935 ~ 2.072) | 0.103 |  | 1.935 (1.085 ~ 3.450) | 0.025 |
| Smoking | 1.926 (1.466 ~ 2.532) | < 0.001 |  | 2.062 (1.426 ~ 2.983) | < 0.001 |  | 1.677 (1.105 ~ 2.547) | 0.015 |
| Prior myocardial infarction | 1.074 (0.802 ~ 1.437) | 0.632 |  | 0.976 (0.657 ~ 1.452) | 0.906 |  | 1.172 (0.756 ~ 1.818) | 0.478 |
| Multi-vessel disease | 0.838 (0.600 ~ 1.168) | 0.297 |  | 1.045 (0.658 ~ 1.660) | 0.853 |  | 0.631 (0.387 ~ 1.030) | 0.065 |
| GFR | 0.986 (0.978 ~ 0.994) | < 0.001 |  | 0.980 (0.970 ~ 0.991) | 0.000 |  | 0.995 (0.983 ~ 1.008) | 0.441 |
| Total cholesterol | 0.715 (0.403 ~ 1.266) | 0.249 |  | 0.651 (0.297 ~ 1.428) | 0.284 |  | 0.667 (0.284 ~ 1.569) | 0.354 |
| LDL-C | 1.320 (0.935 ~ 1.865) | 0.115 |  | 1.475 (0.946 ~ 2.301) | 0.087 |  | 1.083 (0.612 ~ 1.916) | 0.785 |
| Non-HDL-C | 1.759 (1.006 ~ 3.078) | 0.048 |  | 1.762 (0.833 ~ 3.728) | 0.138 |  | 2.294 (0.952 ~ 5.530) | 0.064 |
| Log-transferred hsCRP | 1.162 (1.034 ~ 1.305) | 0.011 |  | 1.191 (1.016 ~ 1.396) | 0.031 |  | 1.152 (0.968 ~ 1.373) | 0.112 |
| LVEF | 0.955 (0.940 ~ 0.970) | < 0.001 |  | 0.945 (0.925 ~ 0.966) | < 0.001 |  | 0.966 (0.944 ~ 0.989) | 0.003 |
| Use of statins | 1.971 (1.408 ~ 2.758) | < 0.001 |  | 1.875 (1.219 ~ 2.884) | 0.004 |  | 2.338 (1.333 ~ 4.103) | 0.003 |
| Tertile of Lp(a) | 1.356 (1.157 ~ 1.590) | < 0.001* |  | 1.336 (1.081 ~ 2.653) | 0.007* |  | 1.400 (1.090 ~ 1.798) | 0.008* |
| Tertile 1 | 1 | / |  | 1 | / |  | 1 | / |
| Tertile 2 | 1.168 (0.854 ~ 1.596) | 0.332 |  | 1.330 (0.880 ~ 2.011) | 0.176 |  | 0.995 (0.608 ~ 1.628) | 0.985 |
| Tertile 3 | 1.828 (1.332 ~ 2.510) | < 0.001 |  | 1.786 (1.168 ~ 2.731) | 0.007 |  | 1.925 (1.175 ~ 3.154) | 0.009 |
| Female | 1.927 (1.377 ~ 2.695) | < 0.001 |  | 2.000 (1.302 ~ 3.067) | 0.002 |  | 1.869 (1.064 ~ 3.279) | 0.030 |
| Age | 1.029 (1.014 ~ 1.045) | < 0.001 |  | 1.022 (1.001 ~ 1.043) | 0.040 |  | 1.042 (1.018 ~ 1.068) | 0.001 |
| Body mass index | 1.013 (0.972 ~ 1.057) | 0.535 |  | 1.019 (0.964 ~ 1.078) | 0.499 |  | 1.011 (0.947 ~ 1.081) | 0.735 |
| Hypertension | 0.649 (0.494 ~ 0.852) | 0.002 |  | 0.613 (0.424 ~ 0.886) | 0.009 |  | 0.709 (0.468 ~ 1.075) | 0.105 |
| Diabetes | 1.496 (1.150 ~ 1.947) | 0.003 |  |  |  |  |  |  |
| History of dyslipidemia | 1.503 (1.089 ~ 2.075) | 0.013 |  | 1.386 (0.931 ~ 2.064) | 0.108 |  | 1.913 (1.074 ~ 3.406) | 0.028 |
| Smoking | 1.933 (1.470 ~ 2.541) | < 0.001 |  | 2.050 (1.418 ~ 2.964) | < 0.001 |  | 1.711 (1.125 ~ 2.601) | 0.012 |
| Prior myocardial infarction | 1.074 (0.803 ~ 1.437) | 0.631 |  | 0.980 (0.658 ~ 1.458) | 0.919 |  | 1.209 (0.780 ~ 1.875) | 0.396 |
| Multi-vessel disease | 0.836 (0.599 ~ 1.167) | 0.293 |  | 1.039 (0.654 ~ 1.653) | 0.870 |  | 0.633 (0.389 ~ 1.031) | 0.066 |
| GFR | 0.986 (0.978 ~ 0.994) | 0.001 |  | 0.981 (0.970 ~ 0.991) | < 0.001 |  | 0.995 (0.983 ~ 1.008) | 0.471 |
| Total cholesterol | 0.701 (0.396 ~ 1.241) | 0.222 |  | 0.640 (0.292 ~ 1.403) | 0.266 |  | 0.673 (0.287 ~ 1.581) | 0.364 |
| LDL-C | 1.325 (0.938 ~ 1.872) | 0.110 |  | 1.468 (0.941 ~ 2.291) | 0.091 |  | 1.108 (0.627 ~ 1.958) | 0.725 |
| Non-HDL-C | 1.783 (1.019 ~ 3.120) | 0.043 |  | 1.792 (0.847 ~ 3.791) | 0.127 |  | 2.226 (0.924 ~ 5.366) | 0.075 |
| Log-transferred hsCRP | 1.160 (1.034 ~ 1.302) | 0.012 |  | 1.196 (1.021 ~ 1.402) | 0.027 |  | 1.144 (0.961 ~ 1.361) | 0.130 |
| LVEF | 0.954 (0.940 ~ 0.969) | < 0.001 |  | 0.944 (0.923 ~ 0.965) | < 0.001 |  | 0.967 (0.946 ~ 0.990) | 0.004 |
| Use of statins | 1.958 (1.400 ~ 2.740) | < 0.001 |  | 1.879 (1.222 ~ 2.890) | 0.004 |  | 2.262 (1.289 ~ 3.968) | 0.004 |
| Quartile of Lp(a) | 1.212 (1.079 ~ 1.362) | 0.001# |  | 1.231 (1.054 ~ 1.439) | 0.009# |  | 1.020 (1.000 ~ 1.041) | 0.049# |
| Quartile 1 | 1 | / |  | 1 | / |  | 1 | / |
| Quartile 2 | 1.017 (0.707 ~ 1.463) | 0.927 |  | 1.024 (0.632 ~ 1.660) | 0.923 |  | 1.061 (0.604 ~ 1.867) | 0.836 |
| Quartile 3 | 1.163 (0.808 ~ 1.674) | 0.416 |  | 1.330 (0.822 ~ 2.152) | 0.246 |  | 0.988 (0.556 ~ 1.756) | 0.966 |
| Quartile 4 | 1.798 (1.244 ~ 2.598) | 0.002 |  | 1.816 (1.106 ~ 2.981) | 0.018 |  | 1.834 (1.039 ~ 3.237) | 0.036 |

CI, confidence interval; HDL-C, high-density lipoprotein cholesterol; hs-CRP, high-sensitivity C reactive protein; LDL-C, low-density lipoprotein cholesterol; Lp(a), lipoprotein a; LVEF, left ventricular ejection fraction; OR, odds ratio

*P for trend for tertile of Lp(a); #P for trend for quartiles of Lp(a)
