# Supplementary material for: Lipoprotein (a) interactions with cholesterol-containing lipids on angiographic coronary collateralization in type 2 diabetic patients with chronic total occlusion
Source: Cardiovasc Diabetol. 2019 Jun 24;18:82. doi: 10.1186/s12933-019-0888-z (PMC6589890; doi:10.1186/s12933-019-0888-z)
Supplement: Supplementary file 2 — Additional file 2: Table S2. Impact of lipid profile on poor collateralization in patients with and without diabetes. [file 12933_2019_888_MOESM2_ESM.docx]

Table S2 Impact of lipid profile on poor collateralization in patients with and without diabetes.

|  | Quartiles of lipid profile | |  | Overall (n =1284) | | |  | Diabetes (n = 706) | | |  | Non-diabetes (n = 578) | | |
| --- | --- | --- | --- | --- | --- | --- | --- | --- | --- | --- | --- | --- | --- | --- |
|  | Range | n |  | Poor/Good | Adjusted OR (95% CI)^a^ | P value |  | Poor/Good | Adjusted OR (95% CI) ^a^ | P value |  | Poor/Good | Adjusted OR (95% CI) ^a^ | P value |
| Lp(a) | per quartile, mg/dL |  |  |  | 1.234 (1.100 ~ 1.384) | < 0.001# |  |  | 1.253 (1.076 ~ 1.461) | 0.004# |  |  | 1.223 (1.023 ~ 1.463) | 0.028# |
|  | T1 < 10.26 | 312 |  | 97/215 | 1 | / |  | 60/105 | 1 | / |  | 37/110 | 1 | / |
|  | 10.26 ≤ T2 < 19.35 | 340 |  | 120/220 | 1.000 (0.699 ~ 1.431) | 1.000 |  | 75/112 | 1.031 (0.640 ~ 1.661) | 0.900 |  | 45/108 | 1.005 (0.577 ~ 1.752) | 0.985 |
|  | 19.35 ≤ T3 < 40.20 | 329 |  | 135/194 | 1.172 (0.819 ~ 1.677) | 0.386 |  | 94/94 | 1.330 (0.829 ~ 2.134) | 0.237 |  | 41/100 | 1.010 (0.574 ~ 1.778) | 0.972 |
|  | T4 ≥ 40.20 | 303 |  | 153/150 | 1.888 (1.313 ~ 2.714) | 0.001 |  | 94/72 | 1.934 (1.189 ~ 3.146) | 0.008 |  | 59/78 | 1.906 (1.089 ~ 3.334) | 0.024 |
| Total cholesterol | per quartile, mmol/L |  |  |  | 1.560 (1.348 ~ 1.805) | < 0.001# |  |  | 1.589 (1.302 ~ 1.938) | < 0.001# |  |  | 1.554 (1.249 ~ 1.935) | < 0.001# |
|  | T1 < 4.00 | 327 |  | 94/233 | 1 | / |  | 48/108 | 1 | / |  | 46/125 | 1 | / |
|  | 4.00 ≤ T2 < 4.76 | 318 |  | 109/209 | 1.305 (0.903 ~ 1.886) | 0.157 |  | 63/88 | 1.789 (1.052 ~3.042) | 0.032 |  | 46/121 | 1.026 (0.610 ~ 1.724) | 0.923 |
|  | 4.76 ≤ T3 < 5.57 | 327 |  | 145/182 | 2.302 (1.562 ~ 3.392) | < 0.001 |  | 107/92 | 3.359 (1.939 ~ 5.820) | < 0.001 |  | 38/90 | 1.503 (0.849 ~ 2.659) | 0.162 |
|  | T4 ≥ 5.57 | 312 |  | 157/155 | 3.681 (2.342 ~ 5.784) | < 0.001 |  | 105/95 | 3.919 (2.123 ~ 7.234) | < 0.001 |  | 52/60 | 4.807 (2.356 ~ 9.810) | < 0.001 |
| LDL-C | per quartile, mmol/L |  |  |  | 1.460 (1.268 ~ 1.682) | < 0.001# |  |  | 1.496 (1.233 ~ 1.815) | < 0.001# |  |  | 1.431 (1.161 ~ 1.764) | 0.001# |
|  | T1 < 2.40 | 305 |  | 86/219 | 1 | / |  | 45/104 | 1 | / |  | 41/115 | 1 | / |
|  | 2.40 ≤ T2 < 3.07 | 324 |  | 114/210 | 1.309 (0.901 ~ 1.900) | 0.157 |  | 68/95 | 1.658 (0.977 ~ 2.812) | 0.061 |  | 46/115 | 1.131 (0.661 ~ 1.936) | 0.653 |
|  | 3.07 ≤ T3 < 3.53 | 334 |  | 151/183 | 2.290 (1.551 ~ 3.382) | < 0.001 |  | 114/86 | 4.029 (2.321 ~ 6.996) | < 0.001 |  | 37/97 | 1.174 (0.656 ~ 2.101) | 0.590 |
|  | T4 ≥ 3.53 | 321 |  | 154/167 | 2.924 (1.885 ~ 4.535) | < 0.001 |  | 96/98 | 3.031 (1.655 ~ 5.551) | < 0.001 |  | 58/69 | 3.525 (1.833 ~ 6.781) | < 0.001 |
| Non-HDL-C | per quartile, mmol/L |  |  |  | 1.564 (1.348 ~ 1.814) | < 0.001# |  |  | 1.553 (1.277 ~ 1.888) | < 0.001# |  |  | 1.614 (1.282 ~ 2.033) | < 0.001# |
|  | T1 < 2.99 | 326 |  | 96/230 | 1 | / |  | 52/105 | 1 | / |  | 44/125 | 1 | / |
|  | 2.99 ≤ T2 < 3.76 | 339 |  | 104/235 | 1.038 (0.720 ~ 1.497) | 0.841 |  | 60/101 | 1.183 (0.704 ~ 1.987) | 0.526 |  | 44/134 | 0.947 (0.561 ~ 1.596) | 0.837 |
|  | 3.76 ≤ T3 < 4.58 | 323 |  | 160/163 | 2.695 (1.825 ~ 3.978) | < 0.001 |  | 109/85 | 3.030 (1.762 ~ 5.211) | < 0.001 |  | 51/78 | 2.479 (1.403 ~ 4.379) | 0.002 |
|  | T4 ≥ 40.19 | 296 |  | 145/151 | 3.247 (2.051 ~ 5.140) | < 0.001 |  | 102/92 | 3.255 (1.763 ~ 5.901 | < 0.001 |  | 43/59 | 3.940 (1.876 ~ 8.273) | < 0.001 |
| HDL-C | per quartile, mmol/L |  |  |  | 0.914 (0.814 ~1.027) | 0.131# |  |  | 0.894 (0.767 ~ 1.043) | 0.156# |  |  | 0.930 (0.777 ~ 1.114) | 0.433# |
|  | T1 < 0.83 | 296 |  | 141/155 | 1 | / |  | 98/83 | 1 | / |  | 43/72 | 1 | / |
|  | 0.83 ≤ T2 < 0.96 | 286 |  | 106/180 | 0.746 (0.516 ~ 1.076) | 0.117 |  | 65/86 | 0.709 (0.439 ~ 1.146) | 0.160 |  | 41/94 | 0.830 (0.463 ~ 1.490) | 0.533 |
|  | 0.96 ≤ T3 < 1.11 | 368 |  | 127/241 | 0.614 (0.434 ~ 0.870) | 0.006 |  | 79/121 | 0.602 (0.381 ~ 0.949) | 0.029 |  | 48/110 | 0.663 (0.383 ~ 1.150) | 0.144 |
|  | T4 ≥ 1.11 | 334 |  | 131/203 | 0.800 (0.555 ~ 1.151) | 0.229 |  | 81/93 | 0.748 (0.461 ~ 1.213) | 0.239 |  | 50/110 | 0.842 (0.477 ~ 1.486) | 0.842 |
| Triglyceride | per quartile, mmol/L |  |  |  | 1.002 (0.887 ~ 1.132) | 0.972# |  |  | 1.043 (0.886 ~ 1.228) | 0.612# |  |  | 0.955 (0.791 ~ 1.152) | 0.629# |
|  | T1 < 1.12 | 309 |  | 119/190 | 1 | / |  | 68/84 | 1 | / |  | 51/106 | 1 | / |
|  | 1.12 ≤ T2 < 1.53 | 352 |  | 122/230 | 0.870 (0.613 ~ 1.234) | 0.434 |  | 73/112 | 0.827 (0.510 ~ 1.340) | 0.440 |  | 49/118 | 0.883 (0.528 ~ 1.478) | 0.636 |
|  | 1.53 ≤ T3 < 2.04 | 340 |  | 148/192 | 1.154 (0.810 ~ 1.643) | 0.428 |  | 94/94 | 1.135 (0.701 ~ 1.838) | 0.606 |  | 54/98 | 1.142 (0.674 ~ 1.936) | 0.621 |
|  | T4 ≥ 2.04 | 283 |  | 116/167 | 0.901 (0.612 ~ 1.326) | 0.596 |  | 88/93 | 1.023 (0.612 ~ 1.710) | 0.931 |  | 28/74 | 0.739 (0.400 ~ 1.367) | 0.336 |
| CI, confidence interval; HDL-C, high-density lipoprotein cholesterol; LDL-C, low-density lipoprotein cholesterol; Lp(a), lipoprotein a; OR, odds ratio.  aMultiple adjustment for gender, age, body mass index, hypertension, diabetes, dyslipidemia, smoking, prior myocardial infarction, multi-vessel disease, glomerular filtration rate, log-transferred high-sensitivity C reactive protein and left ventricular ejection fraction. *# for trend for quartiles of lipid profile. | | | | | | | | | | | | | | |
